# Management Practices for Methicillin-Resistant *Staphylococcus aureus* Bacteremia by Adult Infectious Diseases Physicians

**DOI:** 10.1093/ofid/ofy093

**Published:** 2018-05-19

**Authors:** Natalie Banniettis, Susan E Beekmann, Philip M Polgreen, Shubhi Kaushik, Stephan Kohlhoff, David Gilbert, John E Bennett, Margaret R Hammerschlag

**Affiliations:** 1 SUNY Downstate Medical Center, Brooklyn, New York; 2 Emerging Infections Network, University of Iowa, Iowa City, Iowa; 3 Providence-Portland Medical Center, Portland, Oregon; 4 National Institute of Allergy and Infectious Diseases, Bethesda, Maryland

**Keywords:** bacteremia, GSSA, MRSA, treatment failure, vancomycin

## Abstract

To assess current use of vancomycin for methicillin-resistant *Staphylococcus aureus* bacteremia, we surveyed adult infectious disease physicians. Most respondents reported personal experience with infections failing to respond to vancomycin despite minimum inhibitory concentration data indicating susceptibility. In a hypothetical case of such an infection, most would change to daptomycin with or without other agents.

As of 2014, methicillin-resistant *Staphylococcus aureus* (MRSA) infections occurred with an estimated overall annual incidence of 22.72 per 100 000 people [1]. Vancomycin is the firstline parenteral antibacterial for the treatment of invasive MRSA infections. MRSA strains with intermediate susceptibility to vancomycin (glycopeptide-intermediate *S. aureus* [GISA]; minimum inhibitory concentration [MIC], 4–8 μg/mL) and high-level resistance (glycopeptide-resistant *S. aureus* [GRSA]; MIC ≥ 16 μg/mL) occur but are relatively infrequent. In contrast, several observational studies report a frequent correlation between vancomycin treatment failures and in vitro MICs at the upper end of the official range of “susceptibility” [2–6].

There are many potential reasons for a failure of vancomycin treatment: source control failure, heteroresistant subpopulations, inadequate dosing with failure to achieve an area under the curve (AUC)/MIC ≥400, suboptimal tissue penetration due to biofilms, slow bactericidal activity, and likely other factors [3, 4]. Vancomycin MICs can also vary considerably with the MIC testing method used [7]. Automated susceptibility platforms are reportedly accurate within ±1 log_2_ dilution to MICs determined by broth microdilution, the gold standard, which complicates differentiating an MIC of 1 vs 2 μg/mL [7, 8]. This potential discrepancy is problematic when vancomycin MIC is erroneously reported as <2 μg/mL for resistant *S. aureus* isolates, also known as undercalling an MIC. The 2011 Infectious Diseases Society of America (IDSA) guidelines on the treatment of MRSA infections describe the limitations of in vitro susceptibility testing and the need for effective alternative therapy in patients with discordance between in vitro and clinical responses [7].

With such uncertainly, we queried adult infectious disease (ID) consultants as to their management decisions in patients with MRSA bacteremia and reported glycopeptide-susceptible *S. aureus* (GSSA) with MICs at the higher end of the Clinical and Laboratory Standards Institute (CLSI) range of susceptibility.

## METHODS

An electronic 12-question survey of adult ID physician members of the Emerging Infections Network (EIN) was conducted from November 18, 2015, to December 18, 2015. The EIN is a provider-based network comprised of IDSA members in the United States, Puerto Rico, and Canada. The EIN is funded through a collaboration of the Centers for Disease Control and Prevention and the IDSA [9]. The survey was developed with input from EIN program staff. The goal was to assess ID physician vancomycin use in patients with higher, but susceptible, MRSA MICs using hypothetical patient situations.

The survey was distributed by e-mail, with 2 reminders at weekly intervals for nonrespondents. Respondents were not required to answer all questions. An opt-out option was provided for physicians who do not manage *S. aureus* infections. Categorical variables were compared using a χ^2^ test or Fisher’s exact test with SAS, version 9.3 (Cary, NC). *P* values <.05 were considered significant.

## RESULTS

At the time of the query, there were 1232 active EIN members with an adult infectious diseases consultative practice, of whom 652 (53%) responded to the survey. Respondents represented all areas of North America: South Atlantic (18%), Mid Atlantic (16%), Pacific (16%), East North Central (15%), West North Central (10%), South Central (10%), New England (8%), and Mountain (5%); as well as Canada (1%) and Puerto Rico (0.2%). Employment of respondents was roughly equally divided between academic institutions, private practice, and hospital/clinic settings.

Thirty-five of the 652 (5.4%) respondents did not treat *S. aureus* infections and were excluded from the remaining analysis. Of the remaining 617 respondents, 41, for a variety of reasons, did not routinely receive vancomycin MIC data on MRSA isolates and were not able to respond to related questions. The final denominator for the management questions was 576 respondents. Five hundred thirty-two of the 576 respondents (92%) felt that MICs should be reported; 30 of 576 (5%) were interested only in the global interpretation of the MIC. The clinical laboratory methods utilized by the respondents’ labs for determining MRSA vancomycin MIC varied: Vitek 38%, Microscan 25%, e-test 18%, broth microdilution 3%, and BD Phoenix 6%.

### Treatment Failure or Discordant Results: In Vitro Susceptibility and Clinical Treatment Failure

The majority of respondents, 408 of 574 (71%), reported caring for patients during the past 12 months with persistent MRSA bacteremia after 6 days of vancomycin therapy despite successful source control and achievement of targeted vancomycin troughs (15–20 μg/mL); 289 of the 574 (50%) reported 2 or more such patients. Of the 408 respondents, 214 recalled that the vancomycin MICs were <2 ug/mL, and 127 that the vancomycin MICs were 2 ug/mL. Only 11 respondents reported MICs of more than 2 ug/mL in the clinical failures. In short, there was a high clinical failure rate despite in vitro “susceptibility.”

### ID Consultants’ Antibiotic Management of MRSA Bacteremia in Hypothetical Drug-Injecting Patients

Infectious diseases consultants were asked to manage a hypothetical patient who injects drugs (IVDU) and presents with tricuspid valve endocarditis. Initial blood cultures were known to be positive for *S. aureus,* with susceptibilities pending. The patient had no drug allergies, renal impairment, or other confounders.

The vast majority of consultants, 509 of 572 (89%), said they would initiate therapy with vancomycin; 29 of 572 (5%) said they would start with daptomycin, 4/572 (0.7%) ceftaroline, 1/572 (0.2%) linezolid/tedizolid, and 1/572 (0.2%) telavancin (Table 1). Twenty-eight of 572 (5%) chose “other” as initial empiric therapy. In an open-text field box, 31/572 (5.4%) said they would add a β-lactam (cefazolin or oxacillin/nafcillin) to vancomycin for initial empiric therapy.

**Table 1.** Selected Treatment Options for Methicillin-Resistant *Staphylococcus aureus* Bacteremia in Patients Injecting Drugs by Day of Therapy and Vancomycin MIC

	Day 1 Empiric, %	Day 2 MIC 2, %	Day 8 MIC 2, %	Day 9 MIC 4, %
Vancomycin	89	37	8	0.7
Vancomycin +	5.4		1.9	0.9
Daptomycin	5	54	63	56
Ceftaroline	0.7	6	13	14
Linezolid/tedizolid	0.2	2	2	2
Telavancin	0.2	0.3	0.9	1
Ceftaroline and Daptomycin			6.3	14
Daptomycin +			2.3	5.7
Ceftaroline +				2.2
Linezolid +				0.2
Ceftaroline and Daptomycin +				1.2
Other	5	1		

Number of respondents per day of treatment varied (range, 570–574).Combination therapy legend:Vancomycin + a β-lactam, ceftaroline, rifampin, or gentamicin; Ceftaroline + linezolid, trimethoprim-sulfamethoxazole, rifampin, gentamicin or telavancin; Ceftaroline and daptomycin+ a β-lactam, linezolid, telavancin, rifampin or gentamicin; Daptomycin + a β-lactam, rifampin, gentamicin, linezolid or trimethoprim-sulfamethoxazole; Linezolid + interferon-γ. Other not specified.

Abbreviation: MIC, minimum inhibitory concentration (in μg/mL).

Within 1–2 days, the blood isolate was reported as MRSA with a vancomycin MIC of 2 μg/mL. The patient was still febrile despite vancomycin. At this point, 310 of 573 (54%) said they would switch to daptomycin, 213 (37%) said they would continue treatment with vancomycin, 32 (6%) said they would switch to ceftaroline, 9 (2%) said they would switch to linezolid/tedizolid, 2 (0.3%) said they would switch to telavancin, and 7 (1%) said they would switch to another alternate therapy.

Six days later, and the IVDU patient was still febrile. The blood cultures from day 4 of vancomycin therapy were positive for MRSA with an MIC of 2 μg/mL. At this point, only 35/570 (6%) said they would continue vancomycin at the same dose, and 13 (2%) said they would continue vancomycin at a higher dose. The majority of respondents said they would change therapy from vancomycin: 360 (63%) said they would switch to daptomycin, 76 (13%) to ceftaroline, 11 (2%) to linezolid/tedizolid, and 5 (0.9%) to telavancin. Sixty-nine consultants (12%) said they would opt for combination therapy (Table 1).

The febrile patient’s blood cultures from day 6 were still positive for MRSA on day 8, and vancomycin MIC was reported on day 9 of vancomycin therapy as 4 μg/mL; 565/574 (98%) consultants said they would discontinue vancomycin, whereas only 4/574 (0.7%) said they would continue vancomycin as sole therapy. Five of 574 (0.9%) continued vancomycin as part of combination therapy. The majority of respondents (56%, 323/574) said they would switch therapy to daptomycin alone with confirmation that the organism remains susceptible in vitro. Respondents with the most ID experience (+25 years) were least likely to switch to daptomycin or continue vancomycin at the same dose and were most likely to switch to ceftaroline or use combination therapy (*P* = .0029).

## DISCUSSION

As of the time of this survey, vancomycin was the preferred drug for treating right-sided endocarditis due to MRSA. Persistent bacteremia despite 6 days of vancomycin therapy was surprisingly common, reported as encountered at least 2 times annually by 50% of respondents. The majority of respondents reported initial vancomycin MICs of 1.5–2 μg/mL with persistent MRSA bacteremia, whereas GISA and GRSA were rarely encountered (MIC > 2 μg/mL, 2%), consistent with prior reports of poor response to therapy at the higher end of the CSLI susceptibility range. The median time to clearance of MRSA bacteremia was 7–9 days; however, IDSA guidelines suggest changing therapy earlier if the MIC is 2 μg/mL [7, 10].

Standard laboratory testing does not attempt to detect *S. aureus* with vancomycin heteroresistance (hGISA). Vancomycin hGISA was not a focus of this survey and was not evaluated by the majority of respondents in this study (78%). It seems unlikely that hGISA would account for the high frequency of vancomycin treatment failures in GSSA infections reported in this study. Furthermore, the association of hGISA infection with an increased risk for persistent bacteremia or treatment failure with vancomycin is not clearly established in the literature [11]. Another factor that may influence the susceptible MICs reported with failure is the usage of the Vitek automated microbial identification system (used by the labs of 38% of respondents), which may undercall an MIC of 2 μg/mL [8]. The variability in vancomycin MICs among available testing methods is problematic as accuracy is necessary for appropriate antibiotic selection.

Vancomycin treatment failure due to tolerant GSSA often motivated the treating physician to switch to daptomycin or another drug or drug combination. Therapy was more varied as the days of bacteremia increased, indicating a lack of consensus on optimal management. Only a small proportion continued vancomycin at the same or a higher dose. The use of daptomycin in vancomycin treatment failure comes with a caveat: elevated MRSA vancomycin MICs have been associated with elevated daptomycin MICs, rendering the latter potentially problematic as alternate therapy, especially after exposure to vancomycin empiric therapy [7, 12].

This study is subject to limitations associated with voluntary surveys where there is no random sampling plus voluntary response and recall bias. Nonetheless, the EIN network of ID physicians is extensive, with more than 1500 members representing all 50 states, different clinical settings, and varying levels of experience [9]. This, along with the excellent response rate of 53%, contributes to generalizability of the results, although it should be noted that the network may not be reflective of ID practice in general.

In conclusion, vancomycin treatment failures appropriately lead to recommendations to switch antibiotic therapy despite in vitro susceptibility to vancomycin. This discordance raises many issues. The CLSI breakpoints are based on the results of tube dilution MICs. The survey results suggest that the breakpoints should be reevaluated and defined by the susceptibility test method employed. In the meantime, it is imperative for microbiology laboratories to be aware of the MIC distribution observed by their respective testing methodology and take this into consideration when reporting susceptibilities.
